# Following the funding trail: Financing, nurses and teamwork in Australian general practice

**DOI:** 10.1186/1472-6963-11-38

**Published:** 2011-02-17

**Authors:** Christopher Pearce, Christine Phillips, Sally Hall, Bonnie Sibbald, Julie Porritt, Rachael Yates, Kathryn Dwan, Marjan Kljakovic

**Affiliations:** 1 Melbourne East General Practice Network, Blackburn, Victoria, Australia; 2 Academic Unit of General Practice and Community Health, Australian National University Medical School, ACT, Australia; 3 Australian General Practice Network, Manuka, ACT, Australia; 4 National Centre for Primary Care Research Development Centre, Manchester University, Manchester, UK

## Abstract

**Background:**

Across the globe the emphasis on roles and responsibilities of primary care teams is under scrutiny. This paper begins with a review of general practice financing in Australia, and how nurses are currently funded. We then examine the influence on funding structures on the role of the nurse. We set out three dilemmas for policy-makers in this area: lack of an evidence base for incentives, possible untoward impacts on interdisciplinary functioning, and the substitution/enhancement debate.

**Methods:**

This three year, multimethod study undertook rapid appraisal of 25 general practices and year-long studies in seven practices where a change was introduced to the role of the nurse. Data collected included interviews with nurses (n = 36), doctors (n = 24), and managers (n = 22), structured observation of the practice nurse (51 hours of observation), and detailed case studies of the change process in the seven year-long studies.

**Results:**

Despite specific fee-for-service funding being available, only 6% of nurse activities generated such a fee. Yet the influence of the funding was to focus nurse activity on areas that they perceived were peripheral to their roles within the practice.

**Conclusions:**

Interprofessional relationships and organisational climate in general practices are highly influential in terms of nursing role and the ability of practices to respond to and utilise funding mechanisms. These factors need to be considered, and the development of optimal teamwork supported in the design and implementation of further initiatives that financially support nursing in general practice.

## Background

While the numbers of general practitioners in Australia are falling, over the last five years there has been a minor boom in general practice nursing [1]. Between 2005 and 2007 practice nurse numbers increased from 4924 to 7824, with nearly 60% of general practices now employing at least one nurse [2]. The increased movement of nurses into Australian general practice follows similar moves in the United Kingdom and New Zealand [3,4] where the benefits of adding nurses to the staffing configuration, ranging from improved patient satisfaction to potential cost savings, have been well described [5].

Funding structures have an impact on nurses, both in roles and numbers. The UK has traditionally had a well established system of nurses provided to general practice, initially funded through direct subsidies. The introduction of the Quality Outcomes Framework (QOF) in the United Kingdom in 2004 accelerated a trend first initiated by the General Medical Services contract of 1990 to change this landscape. QOF funds general practice according to defined outcomes. There are 80 indicators in 19 clinical domains, and 36 indicators in 5 organisational domains, and many of these indicators are reached with the support of the practice nurse. The QOF has altered both the roles and the self perceptions of nurses [6,7], as they take on more specific tasks, leaving the GP to 'manage' the overall condition.

The US, with its reliance on fee-for service and insurance backed, private care, represents a different set of challenges. The complex system of insurance agencies in North America creates barriers to the employment of nurses in certain roles [8], while leading to a proliferation of defined positions along a continuum from physicians assistant to nurse practitioner. The numbers of all these groups have been increasing [9,10], leading to tensions between the professional groups [11].

In Ontario, Canada, the Family Health Teams and related models of cares - all of which use collaborative models of practice for teams of nurses, doctors, nurse practitioners and other health workers [12]. These models are pioneering a range of reimbursement models for primary care practitioners, including capitation.

Nurses in Australia have been reported to enhance quality through supporting systematised approaches to health care delivery (eg improving infection control, or patient recall systems) and improving clinician-patient communication [13,14]. There is as yet uncertainty about the most effective way to fund practice nurses to achieve these quality health outcomes. General practice funding mechanisms in Australia provide a number of levers that may be used to achieve this purpose, in addition to professional and organisational development or regulatory approaches. This paper explores the impact of current funding mechanisms on the employment and work practices of nurses in general practice in Australia.

The paper begins with a review of general practice financing in Australia, and how nurses are currently funded. We set out three dilemmas for policy-makers in this area: lack of an evidence base for incentives, possible untoward impacts on interdisciplinary functioning, and the substitution/enhancement debate. We then detail the evidence from the Australian General Practice Nurses Study, a three year multi-site, multidisciplinary study of practice nursing in Australia, that may help guide decision-making.

### General practice financing in Australia

General practice in Australia is delivered largely through fee-for-service private practice, underwritten by a government insurer. An amendment added to Section 51 of the Commonwealth Constitution in 1945 empowered the Commonwealth Parliament to make laws relating to medical and dental services, but not in such a way as would constitute civil conscription. In practice, this has meant that doctors are free to arrange their own fee schedules. There are provisions in group practices for GPs to have a common fee-structure without being charged with anti-competitive behaviour. In the traditional, and most prevalent, organisational model, practice nurses are salaried employees of small-business-owner general practitioners.

This traditional model is changing, however. A variety of models of corporate practice now exist in Australia, ranging from large publicly listed companies to practices owned by some GPs who employ others. In some parts of the country, employed GPs are now in the majority. In the Melbourne East GP Network, for example, 60% of GPs are employees, and in such cases, parties removed from the clinical consultation set fees.

In 1975, the Australian Government introduced Medibank (subsequently revised as Medicare in 1984) a universal health insurance program, that set a fixed rebate for consultations and procedures. Medicare in effect provides a funding stream direct to GPs, as in 73% of consultations [15] they forego any fee on top of the government-determined reimbursement for the service, and bill the government directly. When non-medical services such as those provided by practice nurses are included, this figure rises to 78%. For this reason, and because there is a mandatory contribution of 1.5% of taxable income, many patients would not describe Medicare as a system of patient insurance, but rather as a means of funding health care directly. With 85% of Australians visiting general practice annually and over $278.7 M outlays in rebates in the last calendar year [16], government has now become the largest funder of general practice.

As general practice remains private practice, one option for government to influence behaviour is by adjusting the funding stream and focus. It has done so in a variety of ways. Fee-for-service (FFS) remains the most significant activity, but the Medicare benefits schedule has undergone numerous revisions. Items now exist not just for general consultations but also for specific activities - pap smears and immunisations for instance. A range of incentives for complex fee-for-service activities have been introduced from 2004 under the Enhanced Primary Care Program, including items for care planning for chronic disease management, team care arrangements, and comprehensive health assessments for vulnerable subpopulations.

In addition to direct investment in FFS through item numbers, government has increased funding by broadening the funding base to create a blended payment system. There are two arms to this system: Service Incentive Payments (SIP) and Practice Incentive Payments (PIP), both of which function as pay-for-performance incentives. A SIP is a top-up payment for achieving a goal, usually a cycle of care for asthma or diabetes. A PIP is a practice-based payment for meeting specific, practice targets (e.g. providing after-hours care, teaching medical students, and having a quality computerised record system), which can be even further removed from direct patient care. PIP also provides capitation payments to improve practice infrastructure. In rural areas, for example, practices can access a PIP to a maximum of $40,000 to assist with the employment of a nurse [16].

#### Funding practice nurses in Australia

Until 2004, only activities by doctors and optometrists were eligible for Medicare benefits. Under the Enhanced Primary Care Program, item numbers were made available for services delivered by nurses working in general practice, either through the performance of a health assessment or chronic disease management plan in collaboration with a doctor, or through the direct provision of wound care, immunisation or a Pap smear, each of which attracts a nurse-specific FFS rebate under Medicare. The incorporation of nurses into this funding stream has made employment of a nurse less financially prohibitive for practice owners.

### Dilemmas for policy-makers

Policy makers face three dilemmas in trying to identify and respond to the relationship between funding of nurses and general practice outcomes.

*Dilemma #1 *is the poor evidence base to support the notion that any of the incentive measures improve outcomes. A recent systematic review of the literature on funding incentives and multidisciplinary team care found only two published studies that used experimental or quasi-experimental design, with the remainder being descriptive studies; the authors were unable to find firm evidence on whether or not financial incentives of themselves improve health outcomes [17].

*Dilemma #2 *is the need to ensure that policy initiatives achieve their purpose and enhance productive interdisciplinary working in general practices, while not generating detrimental effects or unacceptable opportunity costs. There is little evidence exploring such effects although the potential for them to occur has been acknowledged [18,19]. They may include an unhelpful concentration on funded activities at the expense of other valuable but less defined activities, or damage to previously productive relationships. In the UK, nurses appear to be central to pay-for-performance outcomes, where the Quality Outcomes Framework led to a range of incentives being tied to demonstrated outcomes. However, some rupture of internal practice goodwill has been documented with the sense that nurses are working hard to produce quality outcomes for the practice which financially benefit GPs, but where the bulk of the burden in achieving these is borne by nursing staff [20].

*Dilemma # 3 *is the question of whether to focus on nurses as a means of supplementing a dwindling medical workforce, or as a way of enhancing the comprehensiveness of health care. These two policy foci are not, of course, mutually exclusive; however they require different emphases. If the former is the primary focus of policy makers, they will need to focus on questions of substitution and task transfer, and fund nurses to take on work currently performed by doctors. If improving complementarity, and thereby quality, is the primary focus then policy-makers will need to focus on new models of care delivery and collaborative practice, and on ways of ensuring that health care is monitored and accountable, and that productive teamwork occurs.

## Methods

This study had two components: a cross-sectional study exploring the scope and contextual determinants of nurse roles (Substudy 1); and a twelve-month longitudinal study exploring change in nurse roles and their impact on general practices as organisations (Substudy 2). For substudy 1, multiple data were collected during day-long visits to 25 practices in NSW and Victoria between 2005 and 2006. The diverse dataset was designed to illuminate the relationships between nurse roles and the practice's physical and managerial structure, and perspectives of nurses, managers and GPs on nurse roles (Table 1).

**Table 1:** Description of datasets for rapid appraisal and longitudinal studies

Substudy 1: Cross-sectional study using rapid appraisal (25 practices)		
**Data collected**	**Participants**	**Comments**

Interviews with nurses	36	Mean length 41 minutes (range, 16-69 minutes)

Interviews with doctors	24	Mean length 27 minutes (range, 12-49 minutes)

Interviews with practice managers	22	Mean length 26.5 minutes (range, 14-60 minutes)

Observation of nurse activity	34	51 hours in 25 practices^1^

Photographs of nurse-identified important working sites	35 nurses; 205 photographs	Mean photographs/practice = 9

Maps of practice layout		7 hand-drawn, 18 printed floorplans

Field notes		25

**Substudy 2: Longitudinal study of change in the nurse's role (7 practices)**		

**Data collected**	**Number**	**Comments**

Baseline practice descriptions including genograms, service use patterns, context descriptions	7	Baseline data on nurses' roles in general practices, and practice attitudes to teamwork. Collected during two workshops attended by a GP, manager and nurse from participating practices

Project planning and evaluation documents, with output data	7	These data explored the success of the change in meeting its own goals.

Monitoring interviews with practice staff at implementation and follow-up (at least 6 months after change implementation)	Nurses: 7 during, 6 after change^2^; Managers: 5 after change; Doctors: 2 after change	Data on the impact of the change process on nurse role(s) from the perspectives of nurses (during and after the change) and managers and doctors (after the change). Practices identified whether a doctor or manager would provide the 12 month interview.

Monitoring interviews with Divisional support staff during implementation and at follow-up (at least 6 months after change implementation)	7 during change; 7 after change	Data explored the impact of the change process on nurse role(s) from the perspective of the external support worker

^1 ^Nurses in one practice had 3 hours of observation

^2 ^Due to staffing turnover, the practice nurse involved in one change project had left at follow-up.

For substudy 2 action research was used to engage practices in a process of collective, internal problem-solving to introduce a change in the role of the nurse. The sampling frame included practices nominated by their Divisions of General Practice as general practices which were early adopters of innovations, and those which were not regarded as early adopters of innovation (1 urban, 3 regional, 3 rural practices in Victoria, NSW, Western Australia, South Australia and Queensland). The impact on the practice was followed with collection of baseline, process and outcome data over one year, and interviews with practice and Division staff (Table 1). Practices received minimal external support from the research team.

### Analysis

Intra-case and inter-case analyses were performed for each practice in both substudies by a multidisciplinary team (sociologist, nurse, GP, policy analyst), probing for emergent themes, using the constant comparison method and cross-checked with practices. Emergent themes included structural elements (health care policy, environment, gender, nursing culture), practice level elements (interprofessional relationships, time-use patterns, spatial structures), and individual factors. One of the key analytical themes was financing as an enabler or barrier to nursing work. This was assessed in terms of scope of nursing activity and degree of nursing autonomy using a two dimensional matrix (shown in table 2).

**Table 2:** Matrix of responsibility delegation to nurses and skillsets indicating funding mechanisms which would support role performance in each quadrant

	Skill Set Used by Nurses		
		Limited	Advanced
	
**Delegation to nurses**	High	Fee for Service items for limited clinical activities	Enhanced primary care items or pay-for-performance in practice with poor team environment
	
	Low	Enhanced primary care items or pay-for-performance for which nurse did not receive specific training	Enhanced primary care items or pay-for-performance in supportive team climate, with training
			Blended payment system Fee-for-service for advanced clinical activities

All data including photographs and floorplans were coded into an NVivo database enabling triangulated data interpretation.

This study was approved by the ANU Human Research Ethics and RACGP Research Ethics Committees. All participants had the research explained to them and gave informed consent.

## Results

### Nursing skillsets and roles

Nurses in Australia undergo a generalist training; there is no dedicated primary care stream. Most of the nurses in this sample had recent experience of working in hospitals. More than half had midwifery qualifications. Others had run a Blood Bank, dialysis units, intensive care and antenatal wards, a regional health enterprise, or worked in health professional education. Only two of the nurses in the sample had come to general practice having worked only in hospitals, with most having worked across three or more parts of the health sector. Most were older women with considerable nursing and life experience and many were among the longest serving members of their practice. These data suggest they had both a broad set of skills, and the capacity to work independently within the general practice team.

Nurses undertook a range of roles within practices that included education, quality control, connectivity and problem solving, as well as the more traditionally recognised role behaviours related to organising and delivering patient care.

#### Fee for service items and nursing activity

Almost all doctors mentioned the FFS items for wound care and immunization and care planning as central planks of their business model for nurses. However, in observations of nurse activity it was clear that these items accounted for only a small proportion of the nurse's time or activity. Twenty per cent of the nurse's time was spent on activity that was directly rebateable under the Medicare Benefits Schedule (MBS) - including helping the doctor with procedures or assessments, for example, as well as the nurse-specific items. When all activities were taken as the denominator, MBS rebateable activity accounted for only 6% of nurse activities. The remainder of their time was spent on a range of other activities including monitoring patients, home visits, quality assurance activities, educating nurses, doctors and other staff members and ongoing outreach to maintain continuity of care between practice doctors.

Many of the activities funded by the MBS, such as wound dressings and immunizations, were activities that nurses were already performing prior to introduction of the item number. The advantages of these items were that they enabled the nurse to have their own identifiable income stream, which reinforced their professional legitimacy within the practice.

"..... the nurses actually now are - well, how do you put it - legally making their income (laughs) because before you were doing dressings or giving immunisations but you couldn't charge for them because the patient hadn't seen the doctor. But now, you know, a lady walks in with her foot this morning and I did a dressing on it and I can use the nurses wound management fees to see her for that." *[PN1, practice 4]*

In general, the inclusion of nurse practice items on the Medicare Benefits Schedule was felt to be important symbolically in underlining the professional presence of nurses as part of the general practice team.

I think we're valued more now probably because the monetary side's a bit better for the doctors. That's being very honest. I think that incentive, I think that's made a big difference because I know a few of them actually say now, well, your area does generate quite a lot of money for the practice because, yeah, which is does which is more so than what it used to. (PN Practice 4)

A broadening and deepening of the scope of clinical activity has occurred, particularly in chronic disease management, where nurses have expanded their patient carer role, enabling them to take more responsibility for their activities. In the following example, the doctor points to the nurse roles of patient carer, organiser and educator, all of which have been brought to the fore through the chronic disease management items.

I think a practice nurse's role in chronic disease management is very valuable because ... they could take the time to educate their patients and to make sure that their patients understand and also to follow them up and do the basic things. Secondly they are also very good, they're better at systematically recalling them and sort of like, you know, just doing that because - and they would be much better in liaising with other people like as far as the coordination's concerned, talking to other allied health practitioners, liaising with services like, you know, the aged care services and things *[GP, practice3]*

We found that among the general practices that had a narrower focus - keeping an eye on specific item numbers rather than expanding the role of the practice nurse through a greater variety of item numbers - collaborative behaviour tended to be controlled by simple business procedures. For example the GP needed to be physically present in the nurse/patient encounter simply to enable the patient to obtain a Medicare rebate. In the following account, a nurse describes the damage to her professional identity by the practice's decision to oversight all nurses; in cases like this there was often a threat to the stability of practice staffing numbers as nurses left to undertake other work.

"I can feel my confidence slipping away because I have to get three people to look at a wound. I have to wait for someone to vaccinate, I can't take a stitch out without somebody having a look at it, and I've been nursing for thirty-five years. And even with the registrar, he's only been out for 12 months and I have to ask him is it all right if I vaccinate someone? So I find that quite belittling and I just find it so antiquated." *[PN2, practice 6]*

This illustrates the importance of underlying attitudes to teamwork in mediating the impacts of funding structures on nurse roles. For nurses, the Enhanced Primary Care (EPC) items in the MBS (health assessments and care planning) are predicated on teamwork between nurses and doctors. In some of the practices, there was significant difficulty taking up EPC items because doctors felt uncomfortable delegating high levels of responsibility to nurses, or "didn't have enough time" to learn how to undertake health assessments or chronic disease planning, or consider how to work in partnership. A number of nurses who felt that their plans to do more complex activities were unsupported by practices, left during the study period to take up other positions outside of general practice. In the case studies, practices with hierarchical organisational structures had more difficulty capitalising on the nurses' contribution to EPC items and could not incorporate them into their business models.

Nurses, managers and doctors reiterated the concern that ongoing expansion of FFS initiatives could paradoxically limit nurses' potential. This is partly because FFS items tended to fund narrow elements of clinical care unless they were carefully constructed, as in the case of the chronic disease management items. The broader PIP incentive to rural practices for a block payment possibly enabled nurses to develop more of their roles; however the extension of this grant to practices with fixed interprofessional hierarchies may not be emancipatory if the practice is unprepared to grant nurses some autonomy over clinical decision-making. Of the 25 practices studied in the first phase of this practice, 24% exhibited styles of working with nurses where she had little autonomy over her own work, and was delegated tasks by doctors.

Key work activities that are unsuited to FFS items and which are currently underfunded elements of nursing work are their work as educators. In interviews, no doctor mentioned the role of nurses in their practice as educators, even though this was very evident in the observation data. Nurses in this study were generally the people who provided pragmatic mentoring for junior doctors and other nurses, and (often in covert ways) education on quality and safety matters for older GPs. This work, like much of their quality and safety work, is conducted in the interstices of other elements of nursing work, and is unfunded.

#### Salary structures of nurses and roles

The salaries of nurses are usually constructed independently of the income stream they might generate. Although nurses can now point to their own income generating capacity, they do not generally receive any direct income from it. All of the nurses in the study were employed. Most practices were owner-operated, but two were owned by universities, and one by a Division. All nurses were paid salaries. Although not all nurses in the study were asked about their rates of pay, there appeared to be some marked differences between practices. Nurses were paid according to state awards for nurses, recommended Australian Medical Association rates or a fee negotiated between the nurse and doctor. Few nurses described negotiating for higher salaries, although many thought they were underpaid. Several left during the course of this study to take up positions in other sectors that were more lucrative and the log of claims that a nursing industrial body was developing during the early part of this study was seen as a threat to the financial viability of the practice.

The money obtained through PIP goes to the practice, and although it may be used to employ and expand nursing roles, nurses are not necessarily involved in the planning. The money can be used as management (or GP principals) see fit, and often there was a sense of frustration from nurses that they were not necessarily seeing the benefits. Several nurses commented on having to arrange (and fund) their own CPD, even though the practice ultimately benefited.

"I was under the impression that all these new changes were about getting practice nurses more involved because they get all this incentive payment for practice nurses. But the practice nurses don't see that money and then they don't utilise us properly. So it's a real sense of frustration." *[PN2, practice 6]*

In this quote, the nurse points to the possibility of some of the PIP scheme payments generated by nurses returning to nurses as a form of top-up incentive. This did not occur in any of the 32 practices in the study

## Discussion

That current nurse funding mechanisms in Australia influence task performance and role structure is clear, but how much, and whether this is in an appropriate direction is uncertain.

This study confirms that nurses undertake a multitude of activities within general practice. That their contribution is valued is evidenced by the significant increase in practice nurses after 2004, when the introduction of nursing item numbers as part of the Enhanced Primary Care Program helped overcome concerns about financial viability. However it is clear that nurses contribute much more to practices than the current targeted funding supports. Fee-for-service items for immunisation and Pap smears have allowed practices to simplify bureaucratic processes that once required doctors to 'stick their heads in' (though there were some practices in our study that still did this) so that patients would be eligible for a Medicare rebate. While this increased some activities in this regard, it also increased concerns that nurse roles in some cases may be limited to tasks that are specifically funded. Rural practices able to access the generalised PIP subsidy were often much less limited in their arrangements.

**Figure 1:**
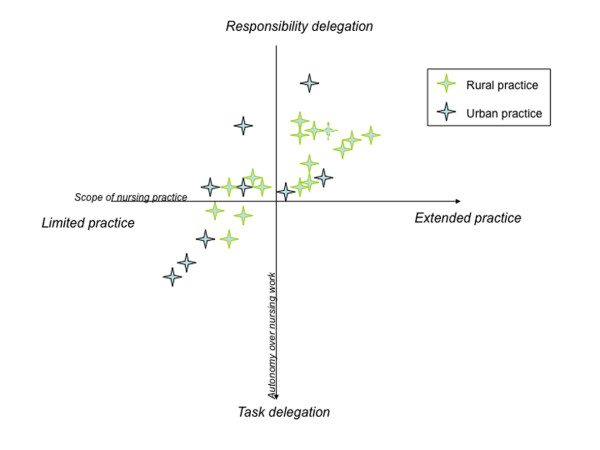
**Organisational factors: Grid of responsibility delegation and skill set for nurses**.

The capacity for different funding models to affect the clinical roles of nurses is moderated by the climate of the general practice. Thus, some hierarchical practices found that they were unable to capitalise on the enhanced skillset of the nurse, because they continued to provide little opportunity for the nurse to have autonomy within the team. The influence of organisational climate on utilisation of funding streams is represented schematically in Figure 1.

In this schema, the upper right quadrant is the domain of optimal roles, where the nurse plies an advanced skillset and has greater autonomy. This zone enables greater adaptation and integration of funding initiatives into practice routines. All of the successful change studies resulted in nurses moving into the upper right quadrant of this model. We documented practices where nurses were only permitted to use the limited FFS items (immunisations, wound care) and were overseen by others (lower left quadrant), and practices where nurses were able to practice their enhanced skills but never in an environment where they had autonomy within the team (top left quadrant). Nurses in the top left quadrant tended to be dissatisfied and perceive the working environment as unstable. In this quadrant, the capacity of enhanced FFS items and PIP items to support the full participation of nurses in the team is undermined by organisational issues. There were few nurses in the upper left quadrant, and these were generally in laissez-faire practices where the nurse was given the responsibility for undertaking services, generally in response to enhanced primary care items, such as care planning, but were not supported to develop an enhanced skillset to meet these tasks.

### Implications for policy

In May 2010, the Australian government allocated $390.3 million from 2012 as payments for general practices to assist them to employ nurses, while removing the FFS items for nurse immunisation and wound management [21].

This study provides some data to help tease out the second policy dilemma discussed in the introduction, and which now face those implementing the new funding policy for nurses: the potential costs of developing particular funding policies for nurses. We have stated that the impact of any funding mechanism is mediated by the team climate in the general practice, and the level of support for nursing autonomy and skill enhancement. As the new grants are outside the FFS structure, they may represent a way of allowing nurses to work more freely and to their scope, but care will need to be taken that doctors are encouraged to move beyond confining hierarchical structures to work in collaboration with nurses. At the same time, practices will need to review their business models to ensure they are able to continue to hire nurses when one element of their reimbursement is removed.

The third policy dilemma - nurses as quality enhancement mechanism or workforce substitute - is answered in our study by suggestion that funding mechanisms can support both approaches. However, if nurses are to work in the optimal zone, research needs to better identify their role in high level FFS areas in general practice as well as undertaking quality enhancement work within a supportive team environment.

Policy makers seeking to enhance the clinical roles of nurses need to explore ways of minimising organisational barriers to enhanced teamwork mediated through extensions to existing funding structures. A risk of creating an open-ended FFS item for nurses may be that in a hierarchical organisational climate, nurses may find themselves overseen and constrained. Conversely, the risk of creating a series of singular, task focused FFS items may be that nursing activity is constrained by a focus on revenue generation and the large proportion of 'unfunded' nursing activity which currently underpins resilience and capacity building for practices, and job satisfaction for nurses, is lost as an opportunity cost. Further changes to the MBS schedule aimed at supporting teamwork need to occur in concert with roll-out activities, possibly through local general practice support organisations (the Divisions of General Practice network), that support interdisciplinary teamwork in practices.

Incentives such as the PIP scheme play to nursing strengths in their capacity to organise systems and their belief in monitoring and benchmarking for quality [13,14]. However, salary structures that do not encourage nurses to benefit from their efforts may alienate nurses and rupture the positive team climate that exists in many general practices. This is a local management issue for general practices, rather than something that can be externally mandated. However, the platform for interprofessional working demands both an accepted salary structure linked to a career path, and the building in of top-up incentives such as those doctors currently receive through the PIP.

Finally, funding is needed to pay for under-recognised nursing activities such as education, which are likely to be central to practice-based education for nurses, doctors, and students into the future, as possibility also mooted for Ontario's Family Health Teams [21]. Because these educational activities do not deal directly with patients, they are difficult to accommodate in a FFS structure, but are important for organisational development and the advancement of truly collaborative teamwork.

### Study limitations

The multimodal, cross-referencing nature of this study is a particular strength. What was observed in practice could be checked with interview material, and comparatively analysed by the research team. We were able, for the seven case studies, to follow a change process in action over a twelve month period. However, the generalisability of this work to all Australian practices is uncertain. Because the research was mainly qualitative, we are unable to test our hypotheses on the relationships between funding, teamwork and organisational resilience. This should be the subject of further research.

## Conclusions

Nurses undertake a range of activities in Australian general practice, not all of which are funded through current FFS payment systems. Under Medicare, FFS payments appear to grow from a policy interest in substitution, with nurses substituting for GP care in specified areas (wound management, immunisation).

This study has demonstrated that interprofessional relationships and organisational climate in general practices are highly influential in terms of nursing role and the ability of practices to respond to and utilise funding mechanisms. Practices with hierarchical workforce arrangements, where GPs are located at the apex, directing and supervising nurses, may not be able to capitalise on funding arrangements like block grants or incentives directed at teamwork. Recent Australian budgetary initiatives to create block grants still reflect an interest in substitution, but recognise that nurses engage in a broader range of quality-enhancing activities. If nurses are to work to their full capacity, there is a need for a parallel program of investment to develop optimal teamwork between the health workers in a practice.

## Competing interests

The authors declare that they have no competing interests.

## Authors' contributions

CP, CP and SY designed the research and drafted the manuscript. All other authors participated in the data analysis and theory development, and contributed to the manuscript. All authors read and approved the final manuscript.

## Pre-publication history

The pre-publication history for this paper can be accessed here:

http://www.biomedcentral.com/1472-6963/11/38/prepub
